# Improvement of the Oxidative Stability of Rosehip, Hemp, and Passion Fruit Oils by the Addition of Monocyclic Phenols as Antioxidants

**DOI:** 10.3390/antiox14030326

**Published:** 2025-03-09

**Authors:** Gloria Castellano, Irene Sarrión, Carmen Fagoaga, Ricardo M. Giménez-Núñez, Enrique Gómez-Gómez, Francisco Torrens

**Affiliations:** 1 Structure-activity Relationship of Bioactive Organic Compounds, Universidad Católica de Valencia San Vicente Mártir, 46001 Valencia, Spain; irene.sarrion@ucv.es (I.S.); carmen.fagoaga@ucv.es (C.F.); riginu@mail.ucv.es (R.M.G.-N.); enrique.gomez@mail.ucv.es (E.G.-G.); 2 Instituto Universitario de Ciencia Molecular, Universidad de Valencia, 46071 Valencia, Spain; torrens@uv.es

**Keywords:** syringic acid, caffeic acid, protocatechuic acid, rosehip oil, hemp oil, passion fruit oil, oxidative stability, antioxidant activity, Rancimat, BQC-Redox System (BRS) system

## Abstract

Caffeic, syringic, and protocatechuic acids are phenolic acids with important antioxidant activity. In this work we studied how the oxidative stability of rosehip, hemp, and passion fruit oils improves with the addition of these antioxidant acids. We used the BQC-Redox System method to measure the antioxidant activity of oils and phenolic acids, and compared these results with other methods described in the literature. In addition, principal components analysis was used to show the relationship between oxidant activity and fatty acids in the oils studied. The results show, in most cases, the improvement of oxidative stability of oils by addition of phenolic acids, and that oxidative stability is determined by the molecular structure of phenolic acids, solubility of oils, the composition of oils in fatty acids, and the influence of temperature in stabilizing phenolic acids and oils. In addition, we support that the BQC-Redox System (BRS) is a suitable method to measure antioxidant activity.

## 1. Introduction

Oxidation of cosmetic oils is a process whereby oils degrade due to a reaction with oxygen, ultraviolet light, high temperatures, and the presence of heavy metals. This can result in the production of free radicals and oxidation products, such as peroxides and aldehydes, which can be harmful to the skin and affect smell, color, texture, and efficacy. Oils composed of more unsaturated fatty acids oxidize faster [1].

Free radicals are known to induce oxidation of nucleic acids, proteins, and lipids, as well as harm intracellular structures including nuclear factor-kappa B (NF-kB), DNA, and activator protein (AP)-1, and up-regulate intracellular transcription protein factors. Protein AP-1 is responsible for the formation of matrix metalloproteinases (MMPs), which crush existing collagen and, consequently, form wrinkles [2]. Preventing oxidation is crucial to maintaining the quality, efficacy, and safety of cosmetic products.

Antioxidants, such as vitamin E (tocopherol), ascorbic acid (vitamin C), BHT (butylated hydroxytoluene), and BHA (butylated hydroxyanisole), can be added to oils to neutralize free radicals and slow down oxidation. However, synthetic antioxidants such as BHA and BHT are also harmful to health, and investigations into new antioxidant products of natural origin that are not harmful and that delay skin aging are required.

Rosehip oil is widely recognized for its regenerative and anti-aging properties, making it a highly valued ingredient in skincare formulations aimed at promoting skin renewal and reducing the visible signs of aging. In contrast, hemp oil is particularly known for its balancing and calming effects, making it an excellent choice for soothing sensitive or irritated skin. Passion fruit oil, on the other hand, is distinguished by its exceptional hydrating and soothing properties, which contribute to maintaining skin moisture and preventing dryness [3,4,5,6,7,8,9,10,11].

A key characteristic of these oils is their high content of unsaturated fatty acids, which, while beneficial for skin health, also make them more susceptible to oxidation. In particular, both rosehip and hemp oils contain significant amounts of polyunsaturated fatty acids (PUFA), which increases their vulnerability to oxidative degradation and reduces their overall stability over time [11,12,13,14,15]. Due to this high PUFA content, these oils require careful storage and handling to minimize oxidation and preserve their beneficial properties.

In comparison, passion fruit oil presents a more moderate oxidation risk. This is attributed to its balanced composition of a number of types of fatty acids. While it is more prone to oxidation than monounsaturated oils, it remains more stable than oils with a predominantly polyunsaturated fatty acid profile. This oxidative stability makes passion fruit oil a versatile option, combining the benefits of hydration and soothing effects with a relatively lower susceptibility to degradation compared to highly polyunsaturated oils.

For all these reasons, the use of plant and antioxidant natural extracts has been encouraged. These include phenolic compounds both monocyclic and flavonoids that are characterized by catching free radicals in the initial stages of oxidation of oils [16,17]. The antioxidant activity of monocyclic phenolic acids depends on their structural moieties, as the number of hydroxyl groups and their ring position in relation to the carboxylic group, and the length of the carboxylic group chain. Phenolic hydroxyl groups are good H-donating antioxidants, which scavenge reactive oxygen species and break the cycle of generation of new radicals. The radical scavenging antioxidants inhibit free-radical-mediated oxidation of lipids, proteins, and DNA, which is implicated in illness [18,19].

Many studies have elucidated the biological activities of flavonoids and phenols, including antioxidants [20], antivirals, anti-inflammatory [21], and antiaging agents [22,23,24,25]. In addition, they reduce the risk of degenerative diseases, coronary diseases, and various types of cancers [26].

In many cases it is argued that natural monocyclic phenolic acids are potent bioactive compounds, but their use as antioxidant agents in lipid-based foodstuffs and cosmetics is limited due to their hydrophilic trait. The low solubility of phenolic acids in nonpolar media limits their bioavailability and functional applications [27,28]. However, caffeic, syringic, and protocatechuic acids are widely used as cosmetic ingredients. Caffeic acid is a hydroxycinnamic acid that exhibits various activities such as emulsifying, ultraviolet-protecting, and radical scavenging [29]. Hydroxycinnamic acids were primarily used in cosmetics as fragrance materials as well as skin and hair conditioners. Nowadays, they are used as active ingredients of cosmetic formulations [30]. Syringic acid, well known for its multilateral anti-inflammatory, antimicrobial, and antioxidant potentials, is used in newly developed linoleic acid transferosomes as an anti-acne nano-form remedy [31]. Protocatechuic acid derivatives were synthesized and the conjugated with alkyl esters to develop effective chemicals that have skin-whitening and antioxidant effects [32].

**Figure 1:**
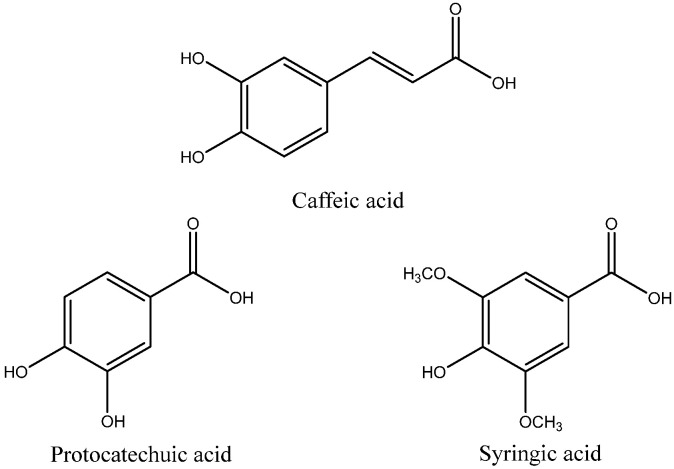
Phenolic acids used as antioxidants for the improvement of the oxidative stability of cosmetics oils.

In this study, we will show how to improve the oxidative stability of rosehip, hemp, and passion fruit cold-pressed oils that are used in cosmetics to avoid skin irritations and produce anti-cancer effects by adding antioxidant caffeic, syringic, and protocatechuic acids (Figure 1). They are phenolic compounds of natural origin and proven antioxidant activity, with different lipophilicity due to molecular characteristics.

To learn the antioxidant activity of phenolic compounds there are several methods that rely on different mechanisms. These include the FRAP (Ferric Reducing Antioxidant Power) method, which measures the compound’s ability to reduce Fe(III) to Fe(II), and the ABTS (2,2′-azino-bis(3-ethylbenzothiazoline-6-sulfonic acid)) method, which measures the ability to donate hydrogen or transfer electrons. Both ABTS and FRAP are widely used methods for assessing the antioxidant capacity of a number of substances, particularly in food science and biochemistry. The FRAP method directly relates to the reducing power of antioxidants, while ABTS is more about free radical scavenging capability.

In addition, this research allows us to compare and/or relate the FRAP and ABTS results obtained in other publications with antioxidant activity with the BQC-Redox system (BRS). The BRS is a device that is used to measure redox parameters in various samples, especially in the agri-food field, such as beverages, plant extracts, and vegetables. It is based on the measurement of total antioxidant capacity (TAC), H₂O₂ scavenging activity, and the removal of reactive oxygen species (ROS) using electrochemical techniques. Methods FRAP and BRS take measurements at room temperature, and the ABTS method at 37 °C.

However, we also research the improvement of the oxidative stability of the oils with phenolic acids with the Rancimat method. Degradation assays on oils at room temperature require long periods of time and are not practical; therefore, accelerated degradation methods with airflow and high temperatures such as Rancimat are becoming more frequent. This method provides an alternative measurement for the oxidation of oils with these phenolic acids that are more sensitive to high temperatures. We can thus check how these antioxidants act at high temperatures and in a lipophilic medium. The Rancimat method accelerates the oxidation process by exposing a fat or oil sample to elevated temperatures (typically 100–150 °C) and a continuous airflow. As the sample oxidizes, volatile acidic compounds (mainly formic acid) are released and carried by the airflow into a measuring vessel containing distilled water. The increase in electrical conductivity in this water indicates oxidation progress and the induction period (IP). The IP is the time before a sharp increase in conductivity and serves as a measure of the oxidative stability of the oils.

Moreover, we will make a principal component analysis (PCA), which gives the relationship of the total antioxidant activity, induction period, and fatty acid composition of the oils used in this research.

## 2. Materials and Methods

Cold-pressed oils: Rosehip. Arganour. Málaga, Spain; Hemp. Ceplame S.L. Valencia, Spain; Passion Fruit. Ceplame S.L. Valencia, Spain.

Composition of cold-pressed oils. The composition of the oils used has been provided by the supplying companies in the previous subsection. The amounts of the components of the oils coincide with those published for Rosehip [4], Hemp [10], and Passion Fruit [33] oils.

Phenolic compounds: Syringic, Caffeic, and Protocatechuic acids. Sigma-Aldrich. St. Louis, MO, USA.

Rancimat—induction period (IP). Oxidative stability of oils was determined by the oxidation induction period (IP) in a 743 Rancimat apparatus (Metrohm Ltd., Herisau, Switzerland) according to the American Oil Chemists’ Society (AOCS) Official Method Cd 12b-92 (AOCS, 1998). Oil samples (3 g) with phenolic compound (0.5% in weight) were heated at 120 °C with a constant airflow of 20 L/h. The time required for a sharp increase in water conductivity was calculated automatically by the software Stabnet 1.1 and corresponds to the induction period in hours. Measurements were taken in quadruplicate.

Statistical analysis. All chemical analyses were carried out in quadruplicate. The results of this study were expressed as means with standard deviations. An Analysis of Variance (ANOVA) was carried out using Real Statistics Resource Pack Software 9.9.1, Challes Zaiontz, and the level of statistical significance was set at *p*-value < 0.05. Principal component analysis (PCA), linear regression correlation, and multiple linear regression model were performed using SPSS (vs. 21.0, IBM Corp., Chicago, IL, USA).

BQC—Redox System (BRS). The BRS device (BQC Redox Technologies, Oviedo, Spain) was used to determine the total antioxidant capacity data. The BRS is a multiparametric portable device based on electrochemistry, designed to measure several REDOX parameters. The BRS gives as data the total antioxidant capacity (TAC), the concentration of Trolox solution with equivalent antioxidant potential to a BRS value of the compound.

The BRS test provides us with the information in a matter of seconds. The Trolox Equivalent Antioxidant Capacity (TEAC) measurements of the phenolic compounds were obtained by taking samples of an electrolyte (BRS)/methanol 1:1 mixture with 0.05% by weight of phenolic compound.

For the cosmetic oils samples of an electrolyte (BRS)/methanol 1:1 mixture with 0.05% by weight of oil were measured.

In the case of the mixtures of cosmetic oils with phenolic compounds, 10 g of oil was mixed with 0.033 g of phenolic acid. Starting from this sample, 0.02 g was dissolved in 400 μL of 1:1 electrolyte/methanol solution. In all cases, they were sonicated with an ultrasound Elmasonic Easy 30 H at 37 kHz and 20 °C. Aliquots of 20 μL have been put in the stick of the BRS device.

## 3. Results

### 3.1. Principal Component Analysis (PCA) of Composition of Cosmetic Oils with Total Antioxidant Capacity (TAC) and Induction Period (IP)

Table 1 shows the composition of the fatty acids (FAs) of cold-pressed cosmetic oils described in the literature.

The antioxidant capacity of these oils measured as TAC, with the BRS-test, and Induction Period (IP) obtained with Rancimat at 120 °C are shown in Table 2. The BRS value indicates the total antioxidant capacity (TAC) of the oils based on electrochemical data. The IP value in hours, obtained by Rancimat, is the time in which the oil oxidizes and, forming peroxide radicals, gives rise to products such as aldehydes, acrolein, epoxides, ethers, and polycyclic aromatic hydrocarbons.

**Figure 2:**
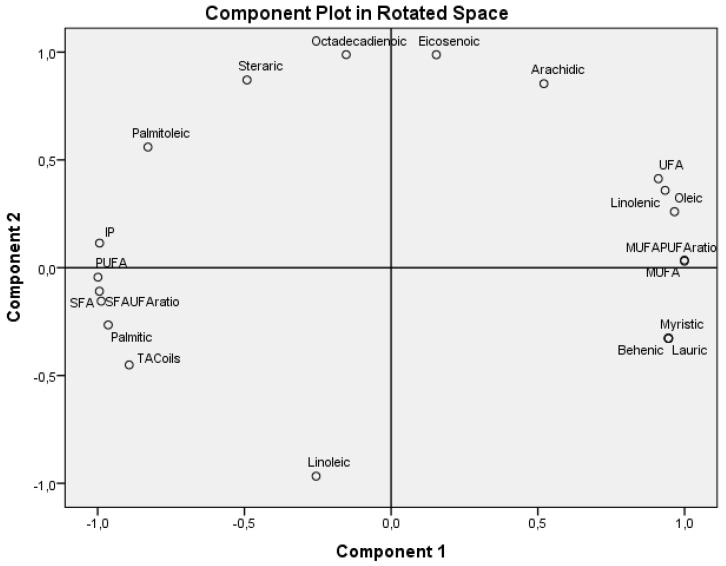
Loading Plot including composition, TAC, and IP of cosmetic oils.

A principal component analysis (PCA) using the composition variables of each of the oils under investigation is included in Table 1. The TAC and IP data included in Table 2 resulted in loading plot Figure 2.

The PCA was applied to reduce the initial variables (Table 1 and Table 2) to a small number of Principal Components (PCs), in order to obtain an overview of the sample variations and identify patterns of behavior. Figure 2 shows a bi-dimensional representation of all variables taken into consideration for the first two PCs. The explained variance by component 1 (PC1) and component 2 (PC2) is 100%. The PC1 explained 71.81% of the total variance, showed positive loading mainly with UFA, MUFA, MUFA/PUFA ratio, myristic, behenic, lauric, oleic, arachidic (all SFAs), linolenic, and eicosenoic acids, and negative loading with IP, TAC oils, SFA, PUFA, SFA/UFA ratio, and stearic, palmitic, palmitoleic, linoleic, and octadecadienoic acids. Component PC2 explained 28.19% of the total variance, was positively correlated with IP, UFA, MUFA, MUFA/PUFA ratio, and palmitoleic, stearic, octadienoic, eicosenoic, arachidic, and oleic acids, and negatively with TAC oils, SFA, PUFA, SFA/UFA ratio, palmitic, myristic, behenic, lauric (all SFAs), and linoleic acids. We can say that IP could be grouped under the PCA with PUFA, SFA, SFA/PUFA ratio, TAC of oils, and palmitic acid, which are in close proximity to each other in Figure 2. This demonstrates the coherence in the relationship between these properties.

### 3.2. Total Antioxidant Capacity (TAC) of Phenolic Acids

The TAC measurements of the phenolic acids used as antioxidants: protocatechuic, caffeic, and syringic acids (Figure 1) obtained with the BRS—Redox System test and literature data are shown in Table 3. Data obtained in the literature of measuring FRAP (antioxidant capacity Fe(III) to Fe(II) reduction) and TEAC-ABTS (Hydrogen or electron donation capacity) used chemical methods.

Table 3 indicates that the results obtained by BRS-values of TEAC, and publications of Rice-Evans [18] and Surco-Laos [19] give similar results. Syringic acid is the most antioxidant phenolic compound, and protocatechuic acid is the least antioxidant.

### 3.3. Effectiveness of Phenolic Acids on the Oxidative Stability of Oils

The oxidative stability of these oils measured as Induction Period (IP) of oils and each oil with each phenolic compound obtained with Rancimat at 120 °C are shown in Table 4. Oxidative stability increases with IP and, therefore, the greater the effectiveness of the added antioxidant phenolic acid.

## 4. Discussion

As shown in Table 2, passion fruit oil (TAC: 1880.88 BRS value, IP: 2.570 h) exhibits the highest total antioxidant capacity when measured using the BRS method, as well as the longest Induction Period (TAC: 85.31 BRS value, IP: 1.212), indicating a greater resistance to oxidation. In contrast, rosehip oil presents the lowest values for both TAC and IP, suggesting a significantly lower oxidative stability. These results highlight the differences in antioxidant capacity and oxidative resistance among the studied oils.

**Figure 3:**
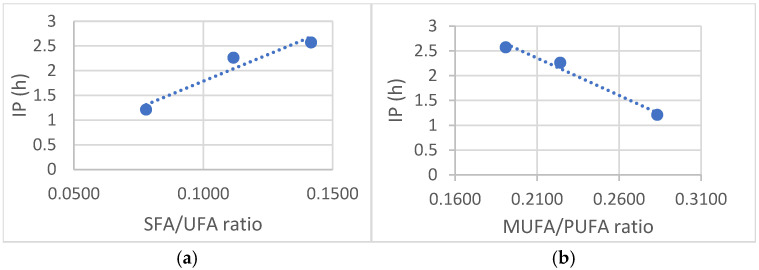
Variation of Induction Period (IP) vs. Total Antioxidant Capacity (TAC): (**a**) SFA/UFA ratio, (**b**) MUFA/PUFA ratio.

Furthermore, Figure 3 illustrates the relationship between the Induction Period and two key parameters: the SFA/UFA ratio (Figure 3a) and MUFA/PUFA ratio (Figure 3b). The observed trend lines indicate a clear correlation between the composition of the oils and their oxidative behavior. Specifically, a higher number of unsaturated bonds in the fatty acid composition of the oil leads to increased susceptibility to oxidation. This suggests that oils rich in polyunsaturated fatty acids, such as rosehip oil, are more prone to oxidative degradation due to their higher degree of unsaturation.

Conversely, the data reveal that an elevated content of saturated fatty acids (SFA) contributes to enhanced oxidative stability. This is particularly evident in the case of passion fruit oil, which demonstrates superior resistance to oxidation compared to oils with a higher proportion of unsaturated fatty acids. These findings reinforce the understanding that the fatty acid composition of an oil plays a crucial role in determining its oxidative stability, with a greater presence of saturated fatty acids providing increased resistance to oxidative processes.

The same results are confirmed in Figure 2 highlighting that oxidative stability (IP) of the oils varies similarly or increases with TAC, PUFA, SFA, SFA/UFA ratio, stearic, octadienoic, palmitic, linoleic, and palmitoleic acids (PC1 < 0), and oppositely IP decreases with MUFA, MUFA/PUFA ratio, eicosenoicarachidic, myristic, lauric, behenic, and linoleic acids (PC1 > 0).

As shown in Table 3, syringic acid exhibits the highest antioxidant capacity among the analyzed phenolic compounds, while compound protocatechuic acid demonstrates the lowest. This result can be attributed to the specific structural characteristics of these compounds, particularly the presence and arrangement of electron-donating groups within their molecular structures.

Syringic acid contains three electron-donating groups (1 hydroxyl and 2 methoxyl) attached to its aromatic ring, which play a crucial role in stabilizing the free radicals formed during oxidation processes. Due to this structural advantage, the radicals generated by syringicc acid exhibit greater stability compared to those formed by caffeic and protocatechuic acids, which each contain only two hydroxyl groups. Since hydroxyl groups act as electron donors, the presence of only two such groups in caffeic and protocatechuic acids results in a lower stabilization capacity compared to syringic acid, which benefits from an additional electron-donating group.

Furthermore, the better behavior of syringic acid in oxidative stability is also influenced by the presence of two ortho methoxy (-OCH_3_) groups in its aromatic ring structure. These methoxyl groups contribute to radical stabilization through their positive mesomeric effect, which arises from the ability of the lone electron pair of oxygen atoms to engage in resonance with an adjacent π-system, such as an aromatic benzene ring or conjugated double bonds. The positioning of these two methoxy groups in the ortho position relative to the hydroxyl group further enhances the antioxidant efficiency of syringic acid. This arrangement increases the overall electron density of the molecule, thereby improving the resonance stabilization of the free radical formed. Consequently, syringic acid is more effective in capturing peroxide radicals generated during the oxidation of oils, prolonging the inhibition of oxidative degradation and reinforcing its role as a highly potent antioxidant.

Caffeic and protocatechuic acids have two hydroxyl groups so they are also antioxidants; however, caffeic acid is more antioxidant than protocatechuic acid because it is derived from cinnamic acid, while protocatechuic acid is derived from benzoic acid. This is explained by the fact that cinnamic acid derivatives are more antioxidant than benzoic acid derivatives, since they have in the aliphatic chain of the aromatic ring a double bond conjugated with the acid group and the aromatic ring that confers greater stability by resonance. The resulting radical of caffeic acid traps free radicals that are generated in the oxidation of the oil.

**Figure 4:**
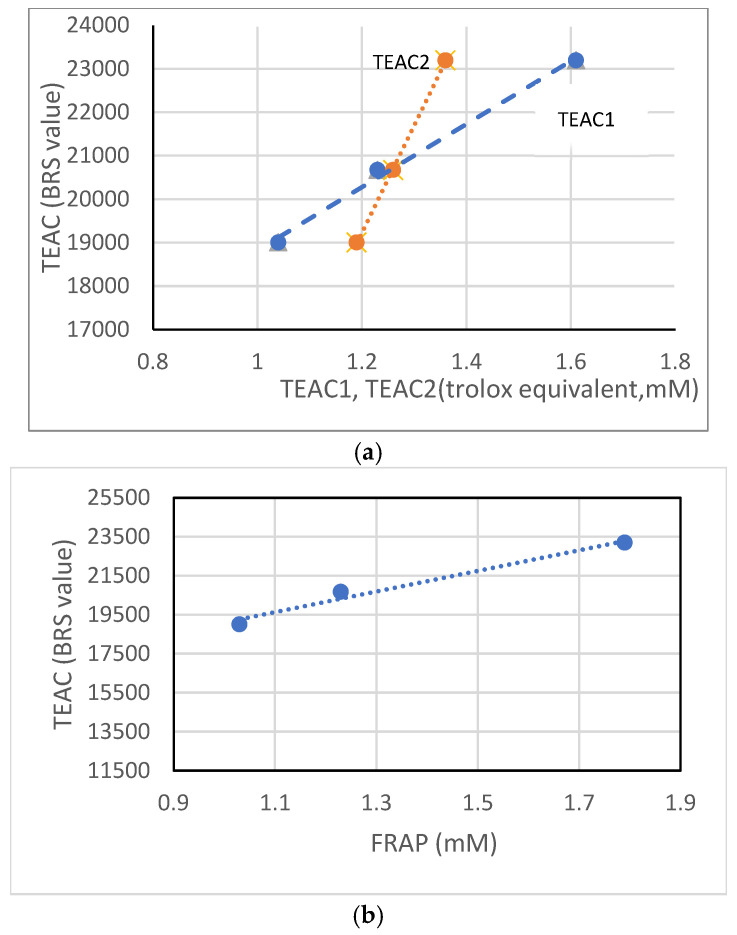
(**a**) Monothone relation TEAC obtained from BRS data vs. TEAC obtained ABTS [18,19] and (**b**) Monothone relation TEAC obtained from BRS data vs. FRAP.

On the other hand, in Figure 4, a monotonic relationship can be observed between the TEAC data obtained using the BRS method and the data reported in previous studies, such as those published by Rice-Evans [18] and Surco-Laos [19] in Figure 4a, as well as with the FRAP data [19] in Figure 4b. This consistency in the results suggests an equivalence between the different methodologies employed for measuring total antioxidant capacity. In particular, the agreement observed between the values obtained using BRS and those from previous studies reinforces the validity of this method as a reliable tool for quantifying antioxidant capacity.

It is important to highlight that, although the BRS method is a relatively recent technique compared to other widely used approaches, the results obtained so far indicate that it is a suitable and reliable method for assessing antioxidant capacity. Its implementation enables precise and reproducible measurements, which contribute to its potential usefulness in scientific studies requiring the evaluation of the antioxidant activity of different compounds.

**Figure 5:**
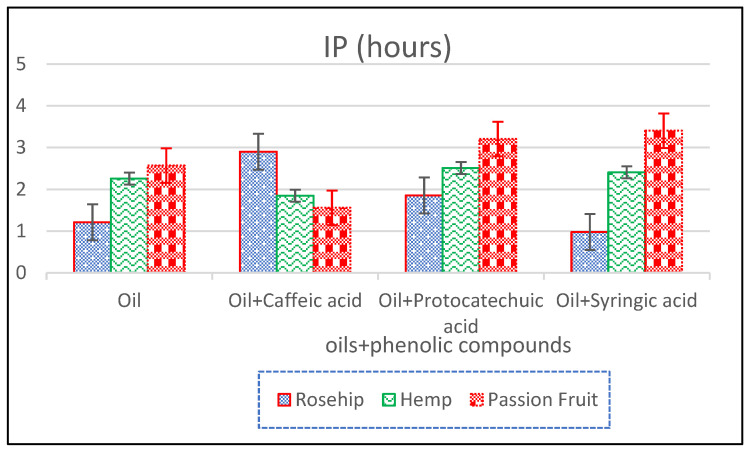
Induction period (IP) in h of oils and oils with phenolic compounds.

As illustrated in Figure 5, the addition of syringic acid led to an increase in the Induction Period (IP) of both hemp and passion fruit oils, while simultaneously reducing the IP of rosehip oil. In contrast, protocatechuic acid exhibited a consistent effect by increasing the IP across all tested oils. On the other hand, caffeic acid displayed a more selective influence, increasing the IP of rosehip oil, while decreasing it in hemp oil and passion fruit oils.

A closer examination of the structural characteristics of these compounds provides insight into their antioxidant behavior. Specifically, compound caffeic acid is a derivative of cinnamic acid (as shown in Figure 1), whereas both protocatechuic and syringic acids are derivatives of gallic acid. Despite caffeic acid exhibiting moderate antioxidant activity, its overall impact on the oxidative stability of oils is not entirely beneficial. In particular, its presence appears to have a destabilizing effect on the hemp and passion fruit oils, potentially reducing their resistance to oxidation. This phenomenon may be attributed to the inherent stability of both oils, which naturally exhibit longer oxidation times. Additionally, caffeic acid is susceptible to decomposition when exposed to high temperatures (120 °C) over prolonged periods, further influencing the oxidative stability of these oils.

In the case of rosehip oil, which inherently possesses a lower IP, the addition of caffeic acid results in a significant enhancement of its oxidative stability. This effect is particularly notable because rosehip oil is exposed to high temperatures for shorter durations due to its lower oxidative stability. Therefore, caffeic acid proves to be an effective antioxidant under conditions of short-term exposure to elevated temperatures, demonstrating its potential for improving the oxidative resistance of certain oils while exhibiting a more complex behavior in others.

Syringic acid improves the oxidative stability of hemp and passion fruit oils (Figure 5). The cause of this is possibly that it has 2 methoxyl groups (Figure 1) so it is more lipophilic and dissolves better in these oils as both have a higher percentage of SFA.

However, syringic acid, being the most antioxidant for the reasons above, does not favor the oxidative stability of rosehip oil, while both caffeic and protocatechuic acids favor it. This may be due to the fact that syringic acid does not act at short IP times, and the other acids, being less antioxidant, do act at low IP.

Although protocatechuic acid has a lower TAC (Table 3), it favors the oxidative stability of all the oils. It is less antioxidant than syringic acid. Therefore, protocatechuic acid favors the oxidative stability of hemp and passion fruit oil to a lesser degree than syringic acid.

## 5. Conclusions

In general, syringic, caffeic, and protocatechuic acids favor the oxidative stability of oils. The oxidative stability of oils varies with their composition. We can affirm that the oxidative stability of the oils is greater with the higher amount of SFAs, lower amount of UFAs (higher SFA/UFA ratio), and the lower the MUFA/PUFA ratio.

Syringic acid has the most antioxidant effect in oils due to the presence of three electron donor groups, specifically two methoxyl groups in ortho with a hydroxyl group, that favor the solubility of this compound in oils, and at the same time, stabilizes by resonance the radical capable of trapping the free radicals that are generated in the oxidation of oils.

On the other hand, the improvement of the oxidative stability of the studied oils depends on the antioxidant activity of the phenolic compounds added due to their different molecular structure. However, other factors must be taken into account such as: (1) solubility of phenolic acids in the oil, which lipolificity is the greater, the higher its saturated fat content; (2) the temperature, in the case of addition of caffeic acid, since at high temperatures in long periods of time, it decomposes, and the oxidative stability of the hemp and passion fruit oils decreases; and (3) the behavior of each antioxidant during the heating process.

Finally, the BRS system is a suitable method to measure antioxidant activity as it has good trend lines with ABTS methods and FRAP. Furthermore, the Rancimat method allows us to know, in an indirect way, the antioxidant capacity of oils and other organic compounds in lipophilic medium and at high temperatures, making it an alternative to ABTS and FRAP. In these two latter methods, when working in a lipophilic medium, modifications to these assays are required to accommodate non-polar (fat soluble) antioxidants. Moreover, they work at room temperature.

## Figures and Tables

**Table 1:** Composition of Fatty Acids in cold-pressed cosmetic oils.

FA/Oil (g/100 g)	Rosehip ^1^	Hemp Oil ^2^	Passion Fruit ^3^
Lauric C12:0	0.04	0.00	0.00
Myristic C14:0	0.07	0.00	0.00
Palmitic C16:0	3.71	6.31	9.73
Palmitoleic C16:1	0.00	0.15	0.11
Stearic C18:0	2.46	2.83	2.58
Octadecadienoic C18:2	2.25	54.85	0.00
Oleic C18:1 n-9	16.12	15.12	13.83
Linoleic C18:2 n-6	41.55	0.52	73.14
Linolenic C18:3 n-3	27.48	18.13	0.41
Araquidic C20:0	0.74	1.03	0.10
Eicosenoic C:20:1	0.44	1.09	0.10
Behenic C:22:0	0.18	0.00	0.00
SFA	7.20	10.17	12.41
UFA	92.42	91.01	87.59
MUFA	20.40	16.36	14.04
PUFA	72.02	72.98	73.55
SFA/UFA ratio	0.08	0.11	0.14
MUFA/PUFA ratio	0.28	0.22	0.19

SFA: Saturated Fatty Acids; UFA: Unsaturated Fatty Acids; MUFA: Monounsaturated Fatty Acids; PUFA: Polyunsaturated Fatty Acids. ^1^ Taken from Jimenez P. et al., 2013 [4]; ^2^ Taken from Özdemir H. et al., 2020 [9]; ^3^ Taken from Malacrida C.R. et al., 2012 [33].

**Table 2:** Oxidative stability measures such as induction period (IP) in hours and Total Antioxidant Capacity (TAC) in cold-pressed oils.

Oil	IP (h)	TAC (BRS Value)
Rosehip	1.212 ± 0.045	85.31 ± 0.22
Hemp	2.257 ± 0.033	540.15 ± 0.12
Passion fruit	2.570 ± 0.010	1880.88 ± 0.14

BRS value: Equivalents of Trolox in an assay of Total Antioxidant Capacity.

**Table 3:** Total Antioxidant Capacities (TEAC) data of phenolic compounds.

Phenolic Compound	Protocatechuic Acid	Caffeic Acid	Syringic Acid
TEAC ^1^(mM)	1.04 ± 0.02	1.23 ± 0.03	1.61 ± 0.01
FRAP ^1^ (mM)	1.03 ± 0.05	1.23 ± 0.07	1.79 ± 0.06
TEAC ^2^ (mM)	1.19 ± 0.03	1.26 ± 0.01	1.36 ± 0.01
TEAC (BRS value)	19,004.64 ± 0.08	20,671.04 ± 0.05	23,194.34 ± 0.03
TAC (BRS value)	30,631.42 ± 0.16	33,145.66 ± 0.14	37,183.03 ± 0.06

TEAC (mM): Trolox equivalent antioxidant capacity assay measured using the ABTS Decolorization Assay; FRAP (mM): Trolox equivalent antioxidant capacity assay measured using the ferric reducing ability of plasma assay; TAC and TEAC (BRS value): Equivalents of Trolox in an assay of Total Antioxidant Capacity with the BQC-Redox System (BRS) device. ^1^ Taken from Surco-Laos, F. et al., 2016 [19]; ^2^ Taken from Rice-Evans, C.A. et al., 1996 [18].

**Table 4:** Induction period (IP) in hours of oils and oils with phenolic compounds.

IP(h)	Oil	Oil + Caffeic Acid	Oil + Protocatechuic Acid	Oil + Syringic Acid
Rosehip	1.212 ± 0.045	2.90 ± 0.08	1.855 ± 0.004	0.980 ± 0.010
Hemp	2.257 ± 0.033	1.85 ± 0.27	2.51 ± 0.04	2.41 ± 0.09
Passion Fruit	2.570 ± 0.010	1.560 ± 0.010	3.205 ± 0.008	3.402 ± 0.008

## Data Availability

The original contributions presented in this study are included in the article. Further inquiries can be directed to the corresponding author.
